# *Cryptococcus neoformans* Secretes Small Molecules That Inhibit IL-1*β* Inflammasome-Dependent Secretion

**DOI:** 10.1155/2020/3412763

**Published:** 2020-12-03

**Authors:** Pedro Henrique Bürgel, Clara Luna Marina, Pedro H. V. Saavedra, Patrícia Albuquerque, Stephan Alberto Machado de Oliveira, Paulo Henrique de Holanda Veloso Janior, Raffael Araújo de Castro, Heino M. Heyman, Carolina Coelho, Radames J. B. Cordero, Arturo Casadevall, Joshua D. Nosanchuk, Ernesto S. Nakayasu, Robin C. May, Aldo Henrique Tavares, Anamelia Lorenzetti Bocca

**Affiliations:** ^1^Laboratory of Applied Immunology, Department of Cellular Biology, Institute of Biological Sciences, University of Brasília, Brazil; ^2^Institute of Microbiology and Infection and School of Biosciences, University of Birmingham, Edgbaston, UK B15 2TT; ^3^Immunology Program, Sloan Kettering Institute, Memorial Sloan Kettering Cancer Center, New York, NY, USA; ^4^Laboratory of Molecular Biology of Pathogenic Fungi, University of Brasilia, Brasilia, Brazil; ^5^Faculty of Ceilândia, University of Brasília, Brazil; ^6^Biological Sciences Division, Pacific Northwest National Laboratory, Richland, Washington, USA; ^7^Bruker Daltonics Inc., Billerica, MA, USA; ^8^Department of Molecular Microbiology and Immunology, Johns Hopkins Bloomberg School of Public Health, Baltimore, MD, USA; ^9^Medical Research Council Centre for Medical Mycology, College of Medicine and Health, University of Exeter and University of Aberdeen, Aberdeen, UK; ^10^Departments of Medicine (Division of Infectious Diseases) and Microbiology and Immunology, Albert Einstein College of Medicine, Bronx, NY, USA

## Abstract

*Cryptococcus neoformans* is an encapsulated yeast that causes disease mainly in immunosuppressed hosts. It is considered a facultative intracellular pathogen because of its capacity to survive and replicate inside phagocytes, especially macrophages. This ability is heavily dependent on various virulence factors, particularly the glucuronoxylomannan (GXM) component of the polysaccharide capsule. Inflammasome activation in phagocytes is usually protective against fungal infections, including cryptococcosis. Nevertheless, recognition of *C. neoformans* by inflammasome receptors requires specific changes in morphology or the opsonization of the yeast, impairing proper inflammasome function. In this context, we analyzed the impact of molecules secreted by *C. neoformans* B3501 strain and its acapsular mutant *Δcap67* in inflammasome activation in an *in vitro* model. Our results showed that conditioned media derived from B3501 was capable of inhibiting inflammasome-dependent events (i.e., IL-1*β* secretion and LDH release via pyroptosis) more strongly than conditioned media from *Δcap67*, regardless of GXM presence. We also demonstrated that macrophages treated with conditioned media were less responsive against infection with the virulent strain H99, exhibiting lower rates of phagocytosis, increased fungal burdens, and enhanced vomocytosis. Moreover, we showed that the aromatic metabolite DL-Indole-3-lactic acid (ILA) and DL-p-Hydroxyphenyllactic acid (HPLA) were present in B3501's conditioned media and that ILA alone or with HPLA is involved in the regulation of inflammasome activation by *C. neoformans*. These results were confirmed by *in vivo* experiments, where exposure to conditioned media led to higher fungal burdens in *Acanthamoeba castellanii* culture as well as in higher fungal loads in the lungs of infected mice. Overall, the results presented show that conditioned media from a wild-type strain can inhibit a vital recognition pathway and subsequent fungicidal functions of macrophages, contributing to fungal survival *in vitro* and *in vivo* and suggesting that secretion of aromatic metabolites, such as ILA, during cryptococcal infections fundamentally impacts pathogenesis.

## 1. Introduction

*Cryptococcus neoformans* is a fungal pathogen that primarily affects immunocompromised patients with acquired immunodeficiency syndrome (AIDS) [1]. *C. neoformans* is responsible for over 180 thousand deaths yearly worldwide [2]. Human infection is usually initiated by the inhalation of environmental spores or yeasts that are present in environmental sources [3–7]. Once in the lung, the fungus is cleared by the host or survives within granulomas. In the context of immunosuppression, primary acquisition or relapse of previously contained yeast can result in disseminated disease, especially involving the central nervous system [5, 8, 9].

The ability of *C. neoformans* to remain viable and survive inside the host is dependent on its many virulence factors, which allow the fungus to modulate and evade the immune response [10, 11]. These virulence factors include enzymes (laccase, urease, phospholipases, proteases, and others) that can be secreted freely or encapsulated in extracellular vesicles [10, 12–14], melanin deposition in the cell wall [14, 15], and the formation of capsular polysaccharides, which are considered the most important of these factors [16–18]. Glucuronoxylomannan (GXM) is the most prevalent of these capsular polysaccharides, facilitating *C. neoformans* resistance against phagocytosis and suppressing various immune responses [19–26]. Altogether, these virulence factors enable this fungus to effectively survive and thrive as a facultative intracellular pathogen, particularly within macrophages [27–33]. Depletion of alveolar macrophages in mice is associated with a worse prognosis during infection with a glucosylceramide mutant, indicating that they might be co-opted by *C. neoformans* during pathogenesis to facilitate fungal growth and dissemination [34]. Phagocytic cells that are unable to kill intracellular yeast cells can return fungal cells to the extracellular environment, either through nonlytic exocytosis called vomocytosis [35–38] or a rapid, highly inflammatory and inflammasome-dependent cell death referred to as pyroptosis [39–41].

The inflammasome is an intracellular multiprotein complex that usually requires an intracellular damage-associated molecular pattern (DAMP) for its oligomerization and proper function [42]. The canonical activation step requires the engagement of an intracellular receptor from the NOD-like receptor (NLR) or AIM2-like receptor (ALR) family, an adaptor protein ASC and the cleavage of procaspase-1. Although some cell types can activate inflammasome pathways from basal expression levels, most of them require extracellular signaling, promoted by membrane-bound pattern-recognition receptors, to initiate inflammasome activation [43]. The activated caspases in this context are responsible for the previously described pyroptosis cell death and, most importantly, for the processing of interleukin- (IL-) 1*β* and IL-18, essential mediators of inflammatory Th1/Th17-driven responses [44].

Among the receptors associated with inflammasome oligomerization, NLRP3 is one of the best described and well characterized in fungal recognition [45]. This receptor is involved in recognition of various fungal species, between yeast and hyphal forms, and opportunistic and primary pathogens [46–51]. The activation of NLRP3 is usually dependent on one or more intracellular stress signals (i.e., potassium efflux; mitochondrial reactive oxygen species production and cathepsin release), which are associated with the interaction between the host cell and the fungus [42, 45]. NLRP3 activation in response to *C. neoformans* only occurs when the yeast is in specific conditions such as biofilms [52], lacking capsule [53], or opsonized before phagocytosis [54]. Moreover, all three classical stress signals are required to activate NLRP3 during these interactions [52]. Notably, mice lacking NLRP3 or ASC are more susceptible to cryptococcal infection with encapsulated yeast cells, whereas infection with acapsular yeast cells results in higher fungal burdens in the lungs in NLRP3 knockout mice [52, 53]. Likewise, susceptibility to cryptococcal infection has also been observed in murine knockout models for IL-1*β* and IL-18 receptors [55, 56].

Different strains of *C. neoformans* elicit variable IL-1*β* induction, especially in *in vitro* models. Although GXM participates in inflammasome inhibition when macrophages are challenged with acapsular strains [53], capsule-independent inhibition of the inflammasome remains poorly understood. Here, we show that other secreted molecules besides GXM can specifically interfere with intracellular signals during inflammasome activation, suppressing various processes associated with this activation and reducing the overall antifungal capacity of macrophages. Furthermore, we have defined two molecules presents in *C. neoformans* conditioned media that participate in inhibiting inflammasome activation in the presence of this remarkable fungus.

## 2. Material and Methods

### 2.1. Ethics Statement

All experimental procedures were approved by the Animal Ethics Committee of the University of Brasilia (UnBDoc number 55924/2016) and conducted according to the Brazilian Council for the Control of Animal Experimentation (CONCEA) guidelines.

### 2.2. Fungal Strains

Cryptococcal species complex strains H99 (var. grubii/*Cryptococcus neoformans*), B3501 (var. neoformans/*Cryptococcus deneorformans*), and *cap67* (acapsular strain derived from B3501) were grown for 18 h in Sabouraud dextrose broth, rotating (120 rpm) at 30°C. Yeast cells were retrieved from culture by centrifugation (5 min, 1800 g) and washed twice in PBS before experiments.

### 2.3. Conditioned Medium, GXM Isolation, and Subsequent Treatments

B3501 and *cap67* strains were grown for 5 days in minimal media (MM) (glucose 15 mM, magnesium sulfate 10 mM, monopotassium phosphate 29.4 mM, glycine 13 mM, and thiamine 3 *μ*M) rotating (120 rpm) at 30°C [57]. Yeast cells were removed from culture by centrifugation (2 × 15 min 5500 g). The supernatant was collected and filtered (0.45 *μ*m) for complete yeast removal. The filtrate was lyophilized and suspended in deionized water, with a tenfold concentration. The products obtained from the B3501 and *Δcap67* strains were denominated conditioned media 35 (CM35) and conditioned media CAP (CMCAP), respectively [58]. CM35 was treated and/or fractioned for subsequent experiments. The size fractions were obtained utilizing an ultrafiltration Amicon system (Millipore), with filtration membranes varying from 100 to 1 kilodalton (kDa). In between fractions (e.g., 100 kDa>CM35>10 kDa) were also obtained, by recovering molecules retained in the filtration membrane. Polarity fractions were obtained by Blight-Dyer technique. Additionally, CM35 was treated by autoclaving (20 min at 123°C) and with the following proteases (24 h at 37°C): trypsin, thermolysin, and pronase. CM35 was also processed to remove GXM using a GXM-capture enzyme-linked immunosorbent assay (ELISA), as described by Rodrigues et al. [59]. Briefly, an ELISA high-binding plate was coated with mAb 18B7 (a monoclonal antibody (Ab) specific for GXM) [60] for 2 h at room temperature, preceded by a blocking step with 1% BSA solution for 1 h. Finally, conditioned media or minimal media were added to the wells for an additional 2 h and recovered at the end. The bound GXM was recovered by elution with Tris-glycine (pH 7.4) buffer. Yeast capsular polysaccharides from B3501 were harvested [61] and kindly supplied by Julie M. Wolf (Albert Einstein College of Medicine). Exopolysaccharides were obtained by the collection of a viscous layer in the 10 kDa membrane during CM35 ultrafiltration, as described [62].

### 2.4. Polysaccharide (PS) Attachment and Immunofluorescence

Polysaccharide (PS) attachment to *Δcap67* cell wall was performed as described [63]. *Δcap67* cells were incubated in yeast-free B3501 conditioned medium (grown in a minimal medium for 4 days) overnight at 37°C. B3501, *Δcap67*, and *Δcap67*-PS were fixed with 4% paraformaldehyde for 30 minutes at room temperature and incubated with PBS+1% bovine serum albumin for 1 h at room temperature. Yeast cells were then incubated with 0.01% Uvitex2B (a chitin marker; Polysciences) and mAb 18B7 (10 *μ*g/ml) for 30 minutes followed by incubation with Alexa Fluor 546 anti-mouse IgG1 (5 *μ*g/ml; Invitrogen) for 30 minutes at 37°C. Cells were then suspended in an antifading agent, mounted on glass slides, and analyzed with a confocal microscope (Leica TCS SP5).

### 2.5. Generation of Bone Marrow-Derived Macrophages (BMDMs and BMMs) and Dendritic Cells (BMDCs)

Bone marrow cells were obtained from C57BL/6 isogenic mice (8–10 weeks old), as previously described [49]. Briefly, femurs and tibias were flushed with RPMI-1640 to harvest the bone marrow cells. Cells were submitted to erythrocyte lysis under a Tris-buffered ammonium chloride solution, seeded (2 × 10^5^ cells/ml), and cultured for 8 days at 37°C in non-tissue culture-treated Petri dishes in 10 ml/dish of RPMI-1640 medium that contained 50 mM 2-mercapto-ethanol. The medium was supplemented with 20 ng/ml murine granulocyte-macrophage colony-stimulating factor (GM-CSF, PeproTech) to obtain BMDCs and BMDMs or 30% conditioned medium from macrophage colony-stimulating factor-secreting L929 fibroblasts (M-CSF) to obtain BMMs. On the third day, an additional 10 ml of a complete medium that contained differentiation-inducing cytokines was added. Half the medium was exchanged on the sixth day of culturing the BMDCs. On the eight-day, non- and loosely adherent BMDCs or firmly adherent BMMs were stripped with TrypLE™ Express (Gibco), harvested, and plated in RPMI-1640 medium supplemented with Fetal Bovine Serum (FBS) and gentamicin.

### 2.6. Murine Cell Culture Interaction with Conditioned Media

BMMs or BMDCs (1 × 10^6^/ml) were incubated at 37°C in a humidified 5% CO^2^ atmosphere. Cells were stimulated with lipopolysaccharide (LPS; 500 ng/ml for 4 h, Sigma-Aldrich), providing the first signal for inflammasome activation. Additionally, cells were incubated for 18 h with or without potential inhibitors: CM35 (and its fractions), CMCAP, minimal medium, glycine (Sigma-Aldrich), glucuronoxylomannan (GXM), and aromatic metabolites: (Sigma) 3-Phenyllactic acid (PLA); DL-p-Hydroxyphenyllactic acid (HPLA) and DL-Indole-3-lactic acid (ILA), alone or in combination. After that, cells were treated with nigericin (20 *μ*M for 40 or 90 minutes, InvivoGen), providing the second signal for inflammasome activation. Alternatively, cells stimulated with LPS were infected with the fungal strains opsonized with mAb 18B7 (a kind gift from A. Casadevall, Johns Hopkins Bloomberg School of Public Health) [60]. Controls included conditions without LPS or nigericin.

### 2.7. Cytokine Quantification by ELISA and Lactate Dehydrogenase (LDH) Detection

The cell-free supernatants of the BMM and BMDC cultures were harvested for measurements of IL-1*β* and tumor necrosis factor- (TNF-) *α* (Ready-Set-Go! Kit, eBioscience) concentrations using ELISA. The determination of intracellular pro-IL-1*β* was performed with the cell lysates (Ready-Set-Go! Kit, eBioscience). The data were expressed as pg/ml ± the standard deviation (SD) of two to three independent experiments, which were conducted in triplicate.

The cell-free supernatants of the BMM cultures were harvested to quantify LDH release, as a cell death marker (CytoTox 96® Non-Radioactive Cytotoxicity Assay, Promega) after treatment with nigericin for 90 minutes. The data were expressed as percentage ± the standard deviation (SD) of two to three independent experiments, which were conducted in triplicate, considering cells without any treatment as 0% and cells treated with 15% dimethyl sulfoxide (DMSO) as 100% of cell death.

### 2.8. Active Caspase-1 Detection by Flow Cytometry

BMMs challenged with *C. neoformans* strains (MOI 5 : 1) opsonized with mAb 18B7 were harvested from the tissue culture well plates with a dissociation agent (TrypLE Express, Thermo Fisher Scientific). 5 × 10^5^ were collected per group before staining. Staining for caspase-1 (FAM-FLICA™ Caspase-1 Assay Kit, Immunochemistry) was performed according to the manufacturer's instructions. The cells were then analyzed in a flow cytometer (FACSVerse, BD Biosciences) with the FITC filter channel (FL-1), and data were processed (FlowJo X, LLC).

### 2.9. cDNA Synthesis and Real-Time Reverse Transcription Polymerase Chain Reaction (RT-PCR)

Total RNA from the cultured BMDMs was extracted using TRIzol reagent (Invitrogen), and cDNA synthesis was performed using a high-capacity RNA-to-cDNA kit (Applied Biosystems), according to the manufacturer's instructions. qRT-PCR was performed using SYBR Green Master Mix and StepOne Real-Time PCR System (Applied Biosystems). The primer sequences were as follows: IL-1*β* forward, 5′-GTGTGTGACGTTCCCATTAGACA-3′; IL-1*β* reverse, 5′-CAGCACGAGGCTTTTTTGTTG-3′; Nf-*κ*B forward, 5′-AGCCAGCTTCCGTGTTTGTT-3′; nuclear factor kappa-light-chain-enhancer of activated B cells (Nf-*κ*B) reverse, 5′-AGGGTTTCGGTTCACTAGTTTCC-3′; GAPDH forward, 5′-TGAAGCAGGCATCTGAGGG-3′; glyceraldehyde 3-phosphate dehydrogenase (GAPDH) reverse, 5′-CGAAGGTGGAAGAGTGGGAG-3′; IL-1*β* or Nf-*κ*B to GAPDH relative expression was calculated using the 2^(-ct)^ method and normalized to the level of unstimulated BMMs.

### 2.10. Vomocytosis Scoring by Flow Cytometry

BMMs challenged with stained H99 with Calcofluor White (10 *μ*g/ml, 10 min, Sigma-Aldrich) were treated concomitantly with CM35 or left untreated [64]. After 2 h of interaction, all wells were gently washed three times with warm PBS. The last wash from the 2 h time point wells were collected, and the cells were lysed. RPMI with fluconazole (10 *μ*g/ml, Sigma-Aldrich) was added in the other wells, and the cells were treated again with CM35 or left untreated. The supernatant and cell lysate were collected in determined time points (6, 12, and 24 h). Right after the collection, both supernatant and lysate samples were centrifuged (2.000 × g, 5 min) for yeast recovery and fixated in paraformaldehyde 4% in PBS until flow cytometry analysis. Samples were accessed by flow cytometry (FACS Fortessa, BD Biosciences), at low-speed acquisition for 4 minutes. Parent yeast cells were detected using a high Calcofluor fluorescence gate. Using the specification provided by the manufacturer, the number of yeast/ml was calculated.

### 2.11. Coincubation Assays (Phagocytic Index, Fungicidal Activity, and Transwell Assays)

BMMs were challenged with H99 opsonized with mAb 18B7 (MOI 2 : 1), and the phagocytosis rates and fungal burdens were analyzed at 37°C. For the phagocytosis assay, BMDMs were primed with LPS (500 ng/ml) for 4 h followed by treatment overnight with conditioned media. Controls were left untreated. After this period, the cells were challenged with opsonized H99. After 2 h, the wells were gently washed three times with warm PBS, and the remaining coculture was fixed and stained with modified Wright-Giemsa stain. The phagocytic index was calculated by multiplying the number of macrophages engaging phagocytosis and the number of yeasts phagocyted per 100 macrophages analyzed. For the yeast intracellular growth assay, BMMs primed with LPS (500 ng/ml) or not were challenged with opsonized H99 and concomitantly treated with conditioned media or left untreated. After 2 h, all the wells were gently washed three times with warm PBS to remove extracellular yeast. Right after the washes as well as 24 h later, macrophages were lysed with 0.05% SDS and intracellular yeasts recovered and measured by colony forming units (CFU) by plating on Sabouraud dextrose agar. The intracellular growth rate was determined by dividing 2 h CFU by 24 h CFU. For the transwell assay, BMMs were seeded in both upper and lower chambers and primed with LPS (500 ng/ml). The BMMs in the lower chamber were challenged with opsonized *Cryptococcus* strains (MOI 5 : 1) or left uninfected, while the BMMs in the upper chamber were not challenged. After 24 h at 37°C, macrophages from the upper chambers were challenged with opsonized green fluorescent protein (GFP) expressing H99 (MOI 2 : 1) and lysed with 0.05% sodium dodecyl sulfate SDS at 24 h postinfection. Alternatively, the lower chambers contained only yeast cells from *Cryptococcus* strains in a media with LPS (500 ng/ml) throughout the essay. The recovered intracellular yeast cells were quantified using flow cytometry.

### 2.12. Detection of GXM Internalization Using Fluorescence Microscopy

BMDMs primed with LPS were treated with CMCAP or CM35 and their fractioned derivatives, as described earlier. BMMs were plated in glass inserts, and after 18 h of interaction with conditioned media, cells were permeabilized and fixed with cold methanol (Vetec) for 10 minutes. After consecutive washes, cells were blocked for 1 h with a 10% FBS solution and stained for GXM with mAb 18B7 (10 *μ*g/ml) as a primary Ab and “Alexa Fluor® 633 Goat Anti-Mouse IgG” (Life Technologies) as a secondary Ab. Cells were also stained with a DAPI solution (Life Technologies). The glass inserts were then recovered, and GXM content internalized by the BMMs was observed under a confocal microscope (Leica TCS SP5).

### 2.13. Gas Chromatography-Mass Spectrometry Analysis

Metabolites from conditioned culture media were derivatized as described [65]. Ketone groups of metabolites were derivatized by adding 20 *μ*l of 30 mg/ml methoxyamine in pyridine (Sigma-Aldrich) and incubating at 37°C for 90 minutes with shaking (1000 RPM). Then, amine, hydroxyl, and carboxyl groups were modified with 80 *μ*l of N-methyl-N-(trimethylsilyl)trifluoroacetamide (MSTFA) (Sigma-Aldrich) with 1% trimethylchlorosilane (TMCS) (Sigma-Aldrich) by shaking (1000 rpm) at 37°C for 30 minutes. Derivatized samples were analyzed in an Agilent GC 7890A using a HP-5MS column (30 m × 0.25 mm × 0.25 *μ*m; Agilent Technologies) coupled with a single quadrupole MSD 5975C (Agilent Technologies). Samples were injected in the splitless mode with the port temperature set at 250°C and oven temperature equilibrated at 60°C. The oven temperature was kept at 60°C for 1 minute and then raised to 325°C at a rate of 10°C/minute and finally finished with 5 minutes hold at 325°C. A fatty acid methyl ester (FAME) standard mix was analyzed with each batch for subsequent retention time calibration purposes. Collected data files were processed with MetaboliteDetector [66], by calibrating retention indices (RI) based on FAME standards and deconvoluting and chromatographically aligning features. Metabolites were identified by matching spectral features and retention indices against a PNNL augmented version of the FiehnLib library containing more than 900 metabolites [67]. Unidentified metabolites were also searched against the NIST14 GC-MS library by comparing spectral features only. All identifications were manually validated. Extracted metabolite intensities were subjected to multivariate data analysis (MVDA) using MetaboAnalyst [68]. The data were median normalized and log transformed followed by principal component, hierarchical cluster, and heat map analysis to identify natural clustering within the data.

### 2.14. *Acanthamoeba* Infection and Treatment with Conditioned Media

*Acanthamoeba castellanii* was cultivated in liquid Yeast Peptone Dextrose Medium (YPD, 2% peptone, 1.8% D-glucose, 0.1% yeast extract) supplemented with 0.0034 M tribasic sodium citrate, 0.004 sodium sulfate, 0.0025% monobasic sodium phosphate, 0.0025% monobasic potassium phosphate, 0.00005% iron ammonium sulfate hexahydrate, and 0.00004% calcium chloride dihydrate at 30°C. *A. castellanii* (5 × 10^5^ cells/ml) and *C. neoformans* H99 (1 × 10^6^ cells/ml) were concomitantly added to culture plates with treatments (10% of the volume): PBS, <1 kDa CM35, minimal medium, or aromatic metabolites (1 mM): ILA alone or in combination with HPLA or with HPLA and PLA. After 24 h, the cells were washed three times with PBS to withdraw any fungi not internalized by amoebas. To lysate the amoebas but not the yeasts, 0.01% Triton X-100 was added, and they were sonicated for 10 minutes. Afterward, the lysate was plated in Sabouraud agar plates, which were incubated for 48 h at 30°C for quantification of CFU.

### 2.15. Murine Infection and Treatment with Conditioned Media

The influence of CM treatment in the host immune response was analyzed using three groups of male C57Bl/6 wild-type mice 8-12 weeks old infected with the virulent strain H99 of *C. neoformans* followed by treatment with <1 kDa CM35 or aromatic metabolites. Each group was formed with eight mice, and all of them were challenged with 8 × 10^4^ cryptococcal cells by intratracheal inoculation. Before the surgery, animals were anesthetized following the instruction of Ethics Committee, injecting a solution of Ketamine-Vetnil and Xylazine-Ceva (1 : 1) in the intramuscular region of one of the hind paws of the mice. The volumes used for anesthesia were according to animal weight. Two and seven days after infection, each group received a treatment of 20 *μ*l of intranasal MM, PBS, 0 <1 kDa CM35, or ILA+HPLA (1 mM). Fourteen days after infection, the mice were euthanized by hypoxia using a CO_2_ chamber, and the lungs and brain were surgically removed to the analysis of fungal burden and cytokine production. For quantification of fungal burden, the tissues obtained were weighed and macerated in 1 ml of PBS using a glass macerator. The homogenized tissues were plated in a Sabouraud agar plate and incubated at 30°C for 48 h, allowing the counting of CFU.

### 2.16. Statistical Analysis

Statistical analysis was conducted using GraphPad Prism v.7.0 software. Data were analyzed by one-way ANOVA followed by Tukey's post hoc test. *P* values less than 0.05 were considered significant. The metabolomics assay was analyzed by *t*-test, PCA, and OPLS, and the metabolite figure was generated from an Excel spreadsheet that was extracted from a heat map using the *t*-test values. For normalization, the data were median centered and log transformed.

## 3. Results

### 3.1. Exopolysaccharide Incorporation on Acapsular Mutant Does Not Impair Macrophage Inflammasome Activation

**Figure 1:**
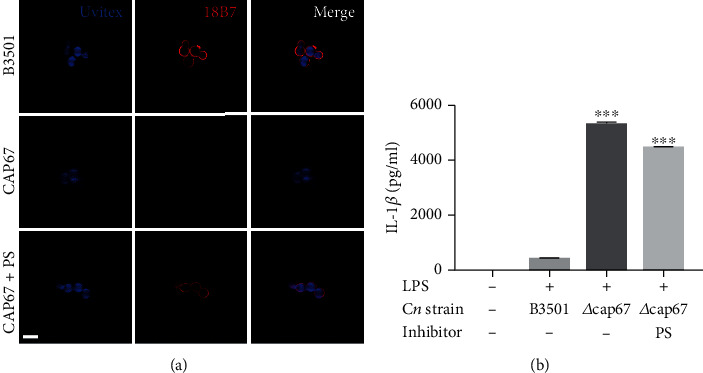
Exopolysaccharide incorporation on an acapsular mutant does not impair macrophage inflammasome activation. (a) GXM detection by immunofluorescence of B3501, CAP67 (*Δcap67*), and CAP67+PS (*Δcap67* coated with polysaccharides from B3501) labeled with Uvitex2B (blue) and mAb against GXM (18B7) (red). (b) BMDMs were stimulated with LPS (500 ng/ml) and infected with opsonized B3501, *Δcap67*, or *Δcap67*+PS strains (MOI 2 : 1) for 24 h. Statistical analysis was performed using one-way ANOVA, where ^∗∗∗^*P* ≤ 0.001. Comparisons were made with the B3501-infected group.

As described previously [53, 54], *C. neoformans* mutants lacking GXM expression and proper capsule formation triggered inflammasome activation more effectively than wild-type encapsulated yeast cells presumably due to the presence of immunomodulatory exopolysaccharides, including GXM, in the fungal capsule. However, we found that *∆cap67* coated with exo-PS which consists mostly of GXM molecules (Figure 1(a)) induced significantly more IL-1*β* secretion compared to the wild-type B3501 (Figure 1(b)). This result suggested that the presence of GXM on the yeast surface by itself was not sufficient to explain the differences in inflammasome activation observed between acapsular mutants and their wild-type counterparts.

### 3.2. CM35, But Not CMCAP or Minimal Media, Reduces IL-1*β* Secretion

**Figure 2:**
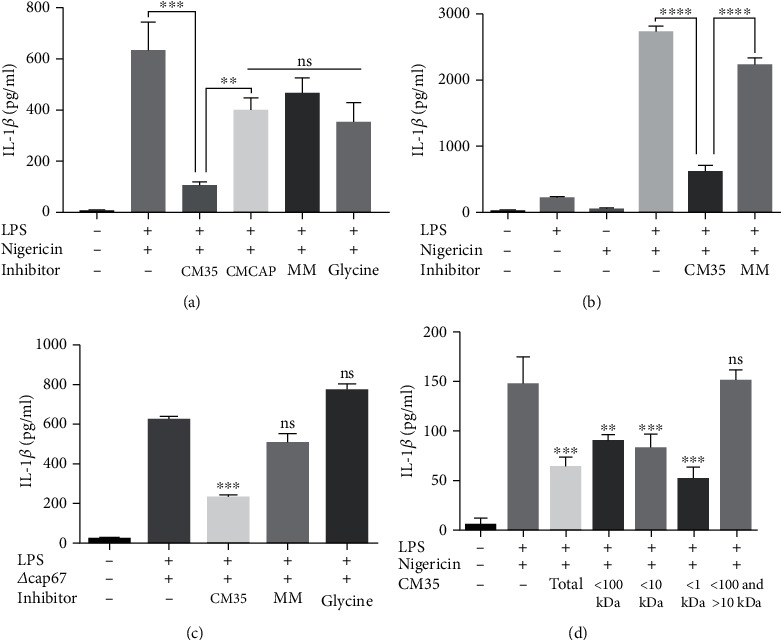
CM35, but not CMCAP or minimal media reduces IL-1*β* secretion. (a) BMMs were stimulated with LPS (500 ng/ml) and/or nigericin (20 *μ*M), added or not by mediums (CM35; CMCAP or MM 10% *v*/*v*) or glycine (13 mM) overnight (18 h). (b) BMDCs were stimulated with LPS (500 ng/ml) and/or nigericin (20 *μ*M), added by media (CM35 or MM 10% *v*/*v*) overnight (18 h). (c) BMDMs were stimulated with LPS (500 ng/ml), infected with *Δcap67* strain (MOI 5 : 1), added or not by mediums (CM35 or MM 10% *v*/*v*) or glycine (13 mM) overnight (18 h). (d) BMDMs stimulated with LPS (500 ng/ml) and/or nigericin (20 *μ*M), added by CM35>100 kDa, <100 and >10 kDa, <10 kDa and <1 kDa (10% *v*/*v*) overnight (18 h). Supernatants were collected after stimulus and cytokines were quantified by ELISA technique. CM35 = conditioned media from B3501; CMCAP = conditioned media from *Δcap67*; MM = minimal media. Statistical analysis was performed utilizing one-way ANOVA, where ns: not significant; ^∗∗^*P* ≤ 0.002; ^∗∗∗^*P* ≤ 0.001. Comparisons were made with the positive control group (LPS+nigericin) when not indicated (c, d).

Since many of the virulence factors presented by *C. neoformans* are secreted [10, 15], we evaluated whether components released by the fungus (CM) were able to inhibit inflammasome canonical activation. The addition of CM35 to activated macrophages (Figure 2(a)) and dendritic cells (Figure 2(b)) significantly reduced the secretion of IL-1*β* by these cells, while CMCAP, MM, and glycine inhibited IL-1*β* secretion to a lesser degree (Figures 2(a) and 2 (b), S1A Fig). Interestingly, CM35, CMCAP, and MM did not reduce the secretion of TNF-*α* (S1B-D Fig), even if added before LPS priming (S1E Fig). The same pattern was observed when conditioned media from H99 strain was used as treatment (S1G-H Fig). The specific interference of conditioned media in the secretion of IL-1*β*, an inflammasome-dependent cytokine, but not in TNF-*α*, an inflammasome-independent cytokine, indicates that the canonical inflammasome pathway is being inhibited. The reduction in IL-1*β* observed with CMCAP and MM is likely explained by the presence of glycine (Figure 2(a)). We tested if the same results were found when using a different stimulus for inflammasome; we primed macrophages with LPS followed by challenging with the acapsular strain *∆cap67*. In these conditions, BMMs still show a marked reduction in IL-1*β* levels in the presence of CM35, while in these conditions MM and glycine did not affect IL-1*β* secretion (Figure 2(c)), indicating that CM35 is able to decrease the inflammasome activation in the context of different activators. To investigate the molecular identity of the component affecting the inflammasome, we further fractionated our media using ultrafiltration devices with nominal molecular weight cutoffs. From CM35, all these molecular weights separated inhibited IL-1*β* secretion with the exception of the fraction where the high molecular weight components were removed (>10 kDa) (Figure 2(d)), indicating that molecules below the 1 kDa range are responsible for inhibition of inflammasome. Since the strongest effect was achieved with the fractions below 1 kDa, the next assays were conducted utilizing this small size fraction (<1 kDa CM35), unless stated otherwise.

### 3.3. <1 kDa CM35 Inhibits Caspase-1 Activation, Promoting pro-IL-1*β* Accumulation and Cell Death Inhibition

**Figure 3:**
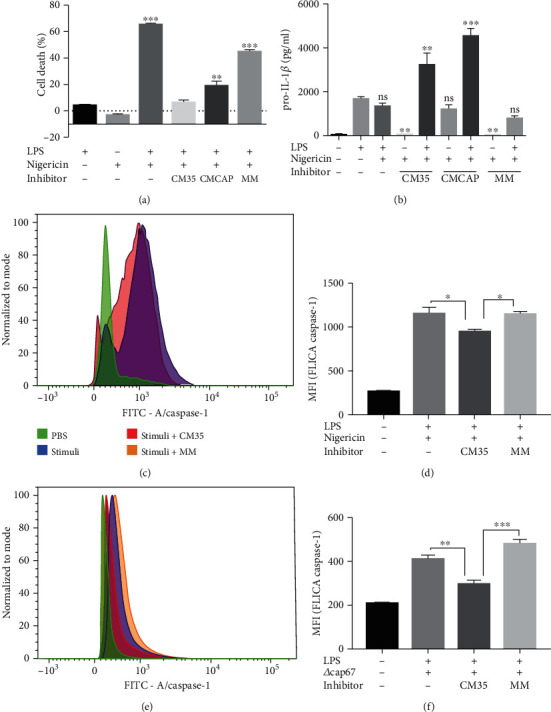
1 kDa CM35 inhibits caspase-1 activation, promotes pro-IL-1*β* accumulation, and inhibits cell death. (a) LDH release from supernatants of BMMs stimulated with LPS (500 ng/ml) and/or nigericin (20 *μ*M), with or without inhibitors (10% *v*/*v*). The medium group was utilized as negative control (0% of cell death) and the DMSO (15%) group was used as positive control (100% of cell death). (b) pro-IL-1*β* production measured from cell lysates of BMMs stimulated with LPS (500 ng/ml) and/or nigericin (20 *μ*M), with or without inhibitors (10% *v*/*v*). (c–f) BMMs stimulated with LPS (500 ng/ml) and nigericin (20 *μ*M) or *Δcap67* (MOI 5 : 1), with or without inhibitors, were analyzed for caspase-1 activation (FLICA). LDH release was measured by colorimetric assay (a), cytokine was measured by ELISA (b), and caspase activation was measured by flow cytometry. (c, d) LPS and nigericin or (e, f) Cap67 was used as stimuli, and the mean fluorescence intensity (MFI) was assessed (d, f). Statistical analysis was performed by one-way ANOVA, where ns: not significant; ^∗^*P* ≤ 0.033; ^∗∗^*P* ≤ 0.002; ^∗∗∗^*P* ≤ 0.001. Comparisons were made with the LPS only group (a, b).

Canonical inflammasome activation triggers various processes in the cell, including, but not limited to, IL-1*β* maturation and secretion. In that context, we analyzed other cell processes related to inflammasome activation, such as intracellular pro-IL-1*β* cleavage and cellular lysis due to caspase-1-mediated pyroptosis. When macrophages were treated with <1 kDa CM35, LDH release was largely abrogated, while treatment with <1 kDa CMCAP or MM resulted in a smaller inhibition of macrophage cell death (Figure 3(a)), which again is likely partly explained by the presence of glycine in the media [69]. <1 kDa CM35 inhibited cell death (as measured by LDH release) following the same pattern seen for IL-1*β* secretion (Figure 3(a)). Regarding pro-IL-1*β*, the results showed that macrophages must be primed with LPS for this cytokine production and that the presence of nigericin does not alter its intracellular levels, indicating LPS-dependent production of the inactive IL-1*β* (Figure 3(b)). When treated with <1 kDa CM35, primed macrophages exhibited an increase in intracellular pro-IL-1*β* protein, while cells treated with MM did not (Figure 3(b)). Surprisingly, treatment with <1 kDa CMCAP also induced a high accumulation of intracellular pro-IL-1*β* (Figure 3(b)). The pro-IL-*β* production might be explained by the fact that <1 kDa CMCAP was the only treatment able to induce pro-IL-1*β* production without LPS. Both conditioned media increased *IL-1β* (i.e., pro-IL-1*β*) transcription levels in primed macrophages, while not similarly inducing *nfκb* transcription, even after 24 h after LPS stimuli (S2 Fig). <1 kDa CMCAP alone was also able to induce TNF-*α* secretion by unprimed macrophages (S1F Fig), corroborating the assumption that this conditioned media can induce first signaling via NF-*κ*B activation.

Having confirmed that <1 kDa CM35 inhibited various signals related to inflammasome activation, the next step was to observe inflammasome activation itself, analyzing the last step in the multiprotein complex assembly: the autocleavage of procaspase-1 into the caspase-1 active form via flow cytometry (Figures 3(c)–3(f)). Results showed that primed macrophages treated with <1 kDa CM35 decreased the caspase-1 active form, compared to untreated cells or cells treated with MM. Moreover, this was independent of nigericin (Figures 3(c) and 3(d)) or infection with *C. neoformans* strains (Figures 3(e) and 3(f)). These results are consistent with the finding that <1 kDa CM35 inhibits IL-1*β* secretion, showing that <1 kDa CM35 can inhibit inflammasome activation induced via different stimuli (i.e., nigericin and infection with *Δcap67*). Altogether, these results indicate that <1 kDa CM35 decreased inflammasome activation, including pyroptotic cell death, pro-IL-1*β* cleavage, the later stages of caspase-1 activation, and consequently IL-1*β* secretion.

### 3.4. <1 kDa CM35 Impacts Phagocytic Capacity and Vomocytosis Events in Interactions between Macrophages and *C. neoformans*

**Figure 4:**
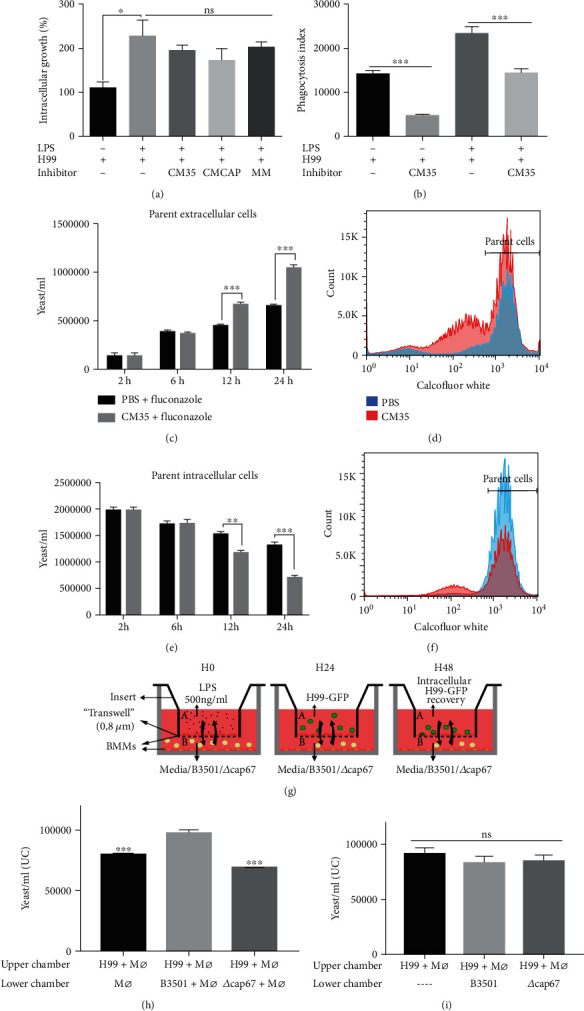
1 kDa CM35 impacts phagocytic capacity and vomocytosis events in interactions between macrophages and *C. neoformans*. (a) Intracellular growth (CFU of 3 h vs. 24 h postinfection) of yeasts cells in BMMs stimulated with LPS (500 ng/ml) and H99 (2 : 1), with or without inhibitors (10% *v*/*v*). (b) Phagocytosis index (2 h postinfection) from BMMs stimulated with LPS (500 ng/ml) and H99 (5 : 1), with or without 1 kDa CM35 (10% *v*/*v*). (c–f) Flow cytometry analysis of (c, d) extracellular and (e, f) intracellular yeast cells (Calcofluor White high 2 h, 6 h, 12 h, and 24 h postinfection) from BMMs infected with H99 (10 : 1), with or without 1 kDa CM35 (10% *v*/*v*). (g) Scheme for transwell infection assay, illustrating the steps taken during the assay. (h, i) Flow cytometry measurement (24 h postinfection) of intracellular yeast cells in BMMs infected with H99 (2 : 1), in the upper chamber of a transwell apparatus. In the lower chambers, BMMs were stimulated with LPS (500 ng/ml) and B3501 or *Δcap67* (5 : 1) (g). Alternatively, only yeast cells from B3501 or *Δcap67* in a media with LPS (500 ng/ml) were included in the lower chambers (h). Statistical analysis was performed utilizing one-way ANOVA, where ns: not significant; ^∗^*P* ≤ 0.033; ^∗∗^*P* ≤ 0.002; ^∗∗∗^*P* ≤ 0.001. Comparisons were made with the B3501 lower chamber infected group (g).

Macrophages are critical for defense against cryptococcosis [70]. We analyzed if >1 kDa CM35 treatment caused a functional impairment of macrophages when infected with *C. neoformans* strain H99. We initially evaluated intracellular fungal burdens in macrophages when yeast cells were added simultaneously with <1 kDa CM35 or minimal media. Interestingly, none of the treatments impacted fungal intracellular growth in primed macrophages (Figure 4(a)). However, when macrophages were primed for 18 h with the conditioned medium, <1 kDa CM35 before infection, we observed a significant reduction in the phagocytic capacity of macrophages (Figure 4(b)).

Another important aspect of macrophage-*Cryptococcus* interaction is vomocytosis. We analyzed vomocytosis in the presence of 1 kDa CM35. Macrophages were concomitantly exposed to <1 kDa CM35, infected with H99 strain and treated with fluconazole, an antifungal drug used for extracellular growth control [29, 71]. We stained H99 cells with Uvitex and allowed macrophages to ingest. If vomocytosis occurs then that would originate highly fluorescent cells in the extracellular space as demonstrated previously. Yeast cells with lower fluorescence were considered to be derived from extracellular growth (daughter cells). We observed that macrophages treated with 1 kDa CM35 had a higher percentage of extracellular parent cells compared to untreated macrophages (Figures 4(c) and 4(d)). The difference between groups starts to increase at 12 h of infection (*P* > 0.001), increasing once again after 24 h while simultaneously intracellular parent yeast cells number showed an inverse pattern, reducing after 12 h of infection in the groups treated with <1 kDa CM35 (Figures 4(e) and 4(f)). Knowing that macrophages treated with 1 kDa CM35 present an equal rate of cell death when compared to untreated cells (Figure 3(a)), the increase of extracellular cells observed in this assay likely indicates an increase in vomocytosis rate in the presence of <1 kDa CM35.

To further study the impact of secreted molecules by *C. neoformans* strains, in macrophage function, a transwell assay involving two sequential infections in different chambers physically separated was carried out (Figure 4(g)). After 24 h, all yeast cells recovered from the upper chamber exhibited high GFP fluorescence, indicating no crossing from yeast cells present in the lower chamber. H99 cells expressing GFP were used for the infection of the macrophages in the upper chamber, while nonfluorescent B3501 or *Δcap67* was used for infection in the lower chamber. A significantly higher intracellular fungal burden was observed in macrophages infected with H99 strain in a chamber vertically adjacent to the bottom chamber containing macrophages infected with B3501 strain compared to fungal burden when the adjacent chamber contained macrophages alone or macrophages infected with *Δcap67* strain or uninfected macrophages (Figure 4(h)). Interestingly, the increase in fungal burden derived by B3501 was not observed when only yeast cells were seeded in the bottom chambers, i.e., the factor produced by B3501 strain is only produced in a stressed milieu, as in the presence of macrophages (Figure 4(i)).

Overall, these experiments indicate that <1 kDa CM35 secreted by B3501 alters macrophage function such that macrophage antifungal activity is affected, potentially by secreting molecules that inhibit a proinflammatory environment.

### 3.5. <1 kDa CM35 Inhibition Characteristics Indicate That a Small, Polar, and Nonpolysaccharide Molecule Is Responsible for the Effects

**Figure 5:**
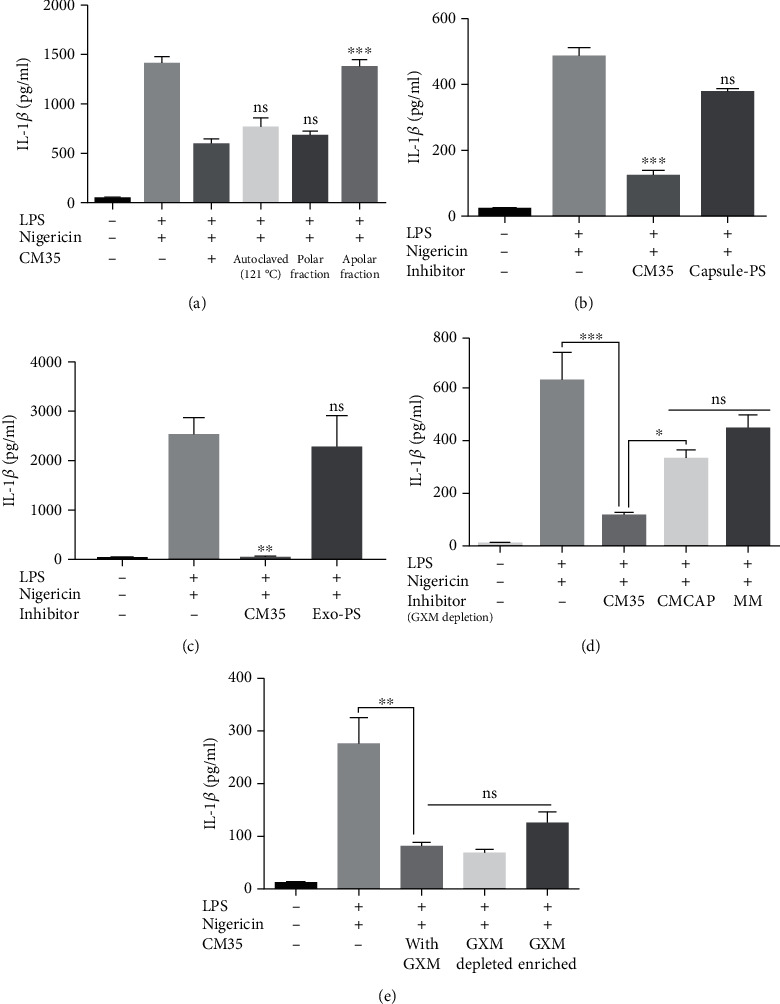
1 kDa CM35 inhibition characteristics indicate a small, polar molecule that does not derive from a polysaccharide origin. (a) BMMs were stimulated with LPS (500 ng/ml) and nigericin (20 *μ*M), with or without 1 kDa CM35 fractioned by water affinity or autoclaved (10% *v*/*v*) overnight (18 h). (b, c) BMMs were stimulated with LPS (500 ng/ml) and nigericin (20 *μ*M), with or without potential inhibitor (10% *v*/*v*) overnight (18 h). Polysaccharides (200ug/mL) extracted from the (c) capsule or secreted in (d) minimal medium were used as inhibitors. (d, e) BMMs were stimulated with LPS (500 ng/ml) and/or nigericin (20 *μ*M), with or without possible inhibitor (10% *v*/*v*) overnight (18 h). Conditioned medium and minimal media (1 kDa CM35, 1 kDa CMCAP, and MM) were treated for GXM depletion utilizing capture ELISA and used as inhibitors (10% *v*/*v*) (e). GXM recovered from ELISA was mixed with 1 kDa CM35 and utilized as an inhibitor (10% *v*/*v*) (f). Supernatants were recovered after stimuli and IL-1*β* secretion was quantified using ELISA. Statistical analysis was performed by one-way ANOVA, where ns: not significant; ^∗^*P* ≤ 0.033; ^∗∗^*P* ≤ 0.002; ^∗∗∗^*P* ≤ 0.001. Comparisons were made with the (a) 1 kDa CM35-treated group or the (b, c) positive control group (LPS+nigericin).

To further characterize the conditioned medium, <1 kDa CM35 was submitted to autoclaving and separated into polar and nonpolar soluble fractions. Autoclaved and polar fraction of CM35 retained its inhibitory properties (Figure 5(a)). Crude <1 kDa CM35 was also treated with various proteases before its addition to primed macrophages. None of the treatments eliminated the inhibition promoted by the conditioned media, and the proteases themselves also did not significantly alter IL-1*β* secretion (data not shown). Overall, the analysis showed that a small, polar, and heat and protease-resistant molecule (or molecules) resulted in the <1 kDa CM35 inhibitory properties.

GXM is a known virulence factor that matches the characteristics possessed by our candidate molecule. While generally depicted as a high molecular weight polysaccharide, this polymer can also exist as lower molecular weight species [72]. We decided to investigate the role of GXM and GXM-derived molecules when interacting with activated macrophages and in the context of <1 kDa CM35 treatment, i.e., if we could detect these molecules in macrophages treated with CM35 below 1 kDa size fraction, but not if treated with <1 kDa CMCAP (S3 Fig). Firstly, polysaccharides derived from the yeast capsule (Figure 5(b)) or exopolysaccharides obtained from the culture media (Figure 5(c)) were used to treat macrophages primed with LPS and nigericin. None of these treatments were able to significantly reduce IL-1*β* secretion compared to <1 kDa CM35, suggesting that GXM is not enough to promote inflammasome inhibition.

Similarly, depleting GXM from <1 kDa CM35 using mAb 18B7 capture protocol [59] did not alter IL-1*β* secretion by activated macrophages (Figure 5(d)). Supporting the hypothesis that GXM is not the candidate molecule, the recovery of GXM from the coated ELISA plate and enrichment of its content in the <1 kDa CM35 did not significantly affect IL-1*β* secretion (Figure 5(e)). Although GXM may be isolated together with our candidate molecule, these assays indicate that GXM does not affect inflammasome inhibition seen in our model.

### 3.6. DL-p-Hydroxyphenyllactic Acid (HPLA) and DL-Indole-3-Lactic Acid (ILA) Participate in Inflammasome Inhibition Property Possessed by <1 kDa CM35

**Figure 6:**
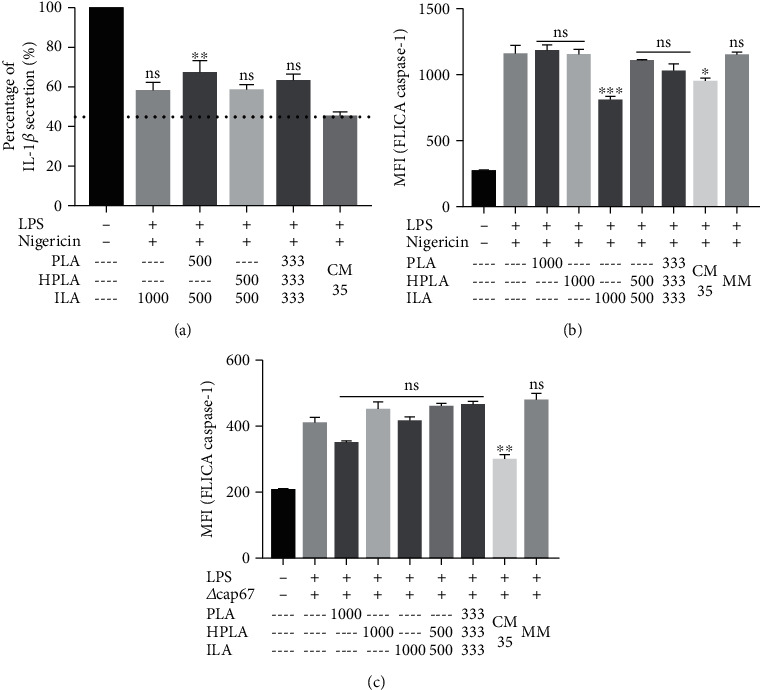
ILA is partly responsible for the inhibitory activity of 1 kDa CM35. (a, b) Percentage of cytokine secretion, measured by IL-1*β* (a) release by BMMs stimulated with LPS+nigericin and the addition of possible inflammasome inhibitors (CM35, PLA, HPLA, and ILA). Positive group LPS+nigericin was normalized for a 100% cytokine secretion. The numbers below the bars represent the concentration of the respective metabolite in *μ*M, except for 1 kDa CM35 and MM (10% *v*/*v*). The graph shows the metabolites alone, followed by double combinations and a triple combination of all metabolites. The bars represent three independent assays. (b, c) BMMs stimulated with LPS (500 ng/ml) and nigericin (20 *μ*M) or *Δcap67* (MOI 5 : 1), with or without inhibitors, were analyzed for caspase-1 activation (FLICA). 1 kDa CM35 = conditioned media from *C. neoformans* B3501; MM = minimal media; PLA = DL-3-Phenyllactic acid; HPLA = DL-p-Hydroxyphenyllactic acid; ILA = DL-Indole-3-lactic acid. Statistical analysis was made utilizing one-way ANOVA, where ns: not significant; ^∗^*P* ≤ 0.033; ^∗∗^*P* ≤ 0.002; ^∗∗∗^*P* ≤ 0.001. Comparisons were made with 1 kDa CM35 group. ns means that the inhibition level achieved by the metabolites is similar to the inhibition achieved by 1 kDa CM35 (a, b). Comparisons were made with the positive control (LPS+nigericin or LPS+*Δcap67*) (c, d).

Given that GXM did not interfere in our inflammasome activation model, we pursued the identification of our active candidate molecule by analyzing <1 kDa CM35 and <1 kDa CMCAP using mass spectrometry. Three candidates met our criteria of being enriched in CM35 vs. CMCAP, small, polar, and heat and protease-resistant molecules: DL-3-Phenyllactic acid (PLA), DL-p-Hydroxyphenyllactic acid (HPLA), and DL-Indole-3-lactic acid (ILA) (S4 Fig). All three are aromatic metabolites derived from amino acid metabolism and are produced by several species of prokaryotes and eukaryotes [73, 74]. Testing these three metabolites separately revealed that ILA, alone or with HPLA, mimicked the activity of >1 kDa CM35 (Figure 6(a)), and ILA alone or associated with HPLA inhibited IL1 *β* secretion and did not affect TNF-*α* secretion (Figure 6(a)). Notably, ILA alone reduced caspase-1 activation to levels similar to that achieved with <1 kDa CM35 (Figure 6(b)). Interestingly, ILA did not reduce caspase-1 activation when macrophages were activated with *Δcap67* infection instead of nigericin (Figure 6(c)). This result suggests that ILA may participate in <1 kDa CM35 inhibition properties, but that it is not the single molecule responsible for this capacity. Hence, it is possible that <1 kDa CM35 inhibitory properties are due to a combination of molecules. At this point, we identify ILA and HPLA as immunomodulatory metabolites produced by *C. neoformans*.

### 3.7. Conditioned Media Also Promotes Survival of *C. neoformans* in *Acanthamoeba castellanii* and Mouse Infection

**Figure 7:**
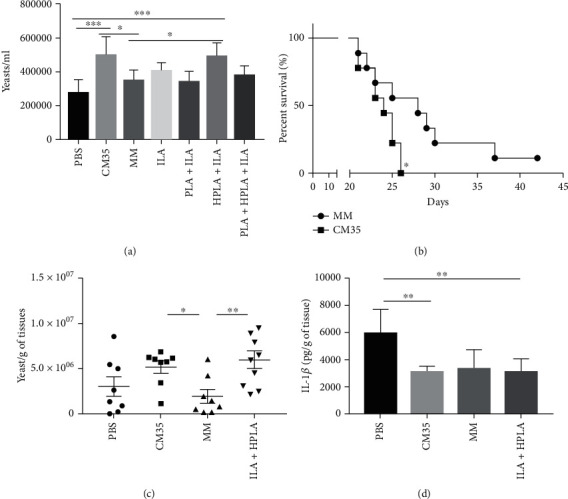
ILA and HPLA increased fungal survival in *Acanthamoeba castellanii* and mice experimentally infected. Intracellular growth (CFU of 3 h vs. 24 h postinfection) inside *A. castellanii* was measured by CFU after treatment with 1 kDa CM35, MM, and ILA associated or not with PLA and HPLA. (b) The survival curve of C57Bl/6 infected with 1 × 10^4^ H99 strain treated with 1 kDa CM35 or MM during each 5 days intranasally. (c) The lung fungal burden from C57Bl/e infected with 1 × 10^4^ H99 strain treated with 1 kDa CM35, MM, or ILA+HPLA each 5 days intranasal repeated after 15 days postinfection. (d) IL-1*β* release in lung tissue after 15 days postinfection from C57Bl/e infected with 1 × 10^4^ H99 strain treated with 1 kDa CM35, MM, or ILA+HPLA each 5 days intranasal repeated. 1 kDa CM35 = conditioned media from *C. neoformans* B3501; MM = minimal media; PLA = DL-3-Phenyllactic acid; HPLA = DL-p-Hydroxyphenyllactic acid; ILA = DL-Indole-3-lactic acid. Statistical analysis was made utilizing one-way ANOVA, where ns: not significant; ^∗^*P* ≤ 0.033; ^∗∗^*P* ≤ 0.002; ^∗∗∗^*P* ≤ 0.001. Comparisons were made with the PBS group (a, b). Comparisons were made with the (c) MM or (d) PBS group.

To evaluate the properties of <1 kDa CM35 in promoting *C. neoformans* survival during infection in other organisms, *A. castellanii* were infected with H99 strain of *C. neoformans* and treated or not with <1 kDa CM35 or aromatic metabolites. The quantification of CFU was performed 24 h postinfection and showed a significantly higher fungal load inside amoebas in the groups treated with <1 kDa CM35 and ILA combined with HPLA, compared to the other groups (Figure 7(a)), indicating that both ILA+HPLA and <1 kDa CM35 enhance fungal survival inside *A. castellanii*. To evaluate if <1 kDa CM35 was effectively favoring *C. neoformans* survival and increasing infection in a more complex host, we treated or not infected mice with <1 kDa CM35 or MM, each 5 days, and analyzed their survival for 45 days (Figure 7(b)). Mice treated with MM survived longer than mice treated with <1 kDa CM35, showing that active metabolites in <1 kDa CM35 enhance infection. Furthermore, when infected C57BL/6 mice were treated with <1 kDa CM35 or ILA plus HPLA at two and seven days postinfection, the fungal load was significantly higher in the lungs of mice treated with <1 kDa CM35 or ILA+HPLA (Figure 7(c)) compared to the lungs of mice treated with PBS or MM at 14 days of infection. Contributing to this result, we found significantly lower levels of IL-1*β* in the lungs of mice treated with <1 kDa CM35 or ILA+HPLA, but not with MM (Figure 7(d)).

## 4. Discussion

The processes by which fungi manipulate the mammalian immune systems and in particular their interaction with inflammasome pathways remain unknown. Although the significant receptors involved and most of the stress signals required for inflammasome activation are known, the mechanisms by which fungal pathogens modulate and/or evade these responses are still poorly understood, when compared to its bacterial counterparts. Here, we demonstrate that molecules secreted by *C. neoformans* can specifically inhibit the canonical activation of the inflammasome pathway and dampen macrophage anticryptococcal activity and mouse immune response, potentiating fungal survival and growth during the host-pathogen interaction. Furthermore, we determined that GXM did not participate in this process, and we identified one molecule partially responsible for the effect promoted by the fungal conditioned media.

Inflammasome activation is associated with a proinflammatory response, mainly due to its IL-1*β* and IL-18 processing properties. Both are critical cytokines for macrophage activation as well as in the development of Th17 and Th1 polarization, respectively [44]. Therefore, inflammasome activation critically affects Th1/Th17 polarization which is widely reported as a protective response against pathogenic fungi. NLRP3 defects are therefore associated with a poor prognosis upon fungal infection, ranging from severe susceptibility in invasive candidiasis [46] to milder susceptibility to cryptococcosis [52]. On the other hand, NLRP3 defects have also been associated with a better prognosis in aspergillosis associated with cystic fibrosis [75], and these defects had no impact in chromoblastomycosis [50]. Hence, inflammasome activation has different effects depending on the fungal pathogen and the associated pathologies of the host.

As the canonical inflammasome scaffold involves various proteins and downstream signaling, numerous steps can be inhibited to prevent scaffold formation, thus preventing the cascade at several steps with different consequences to the cell activation. A single point of interference is sufficient to disrupt the entirety of downstream signaling and inflammasome function, from receptor activation to caspase autolysis [76]. Inflammasome activation can also be partially prevented by the use of cytoprotective agents like glycine [69]. Our study demonstrated that conditioned media from *C. neoformans* strain B3501 promoted robust inhibition of IL-1*β* secretion and complete inhibition of LDH release, although it had less of an impact on caspase-1 activation. IL-1*β* can be secreted by various mechanisms, depending on the cell status, for example, through membrane pores formed by Gasdermin D or through cell membrane defects during pyroptosis [77]. In this model, glycine was not able to prevent pore formation in immortalized macrophages, consequently preventing LDH release but not cytokine secretion. Studies also depicted that dying macrophages undergoing pyroptosis were the primary source of IL-1*β* secretion in an *in vitro* model, with peritoneal macrophages exhibiting caspase-1 activation and a cytokine burst that coincided with the moment of cell death. Interestingly, caspase-1 activation inhibition prevented IL-1*β* secretion, but it did not alter cell death events [78, 79]. Taken together, these findings show that both events can occur independently, even if they are mostly caspase-1 dependent and depend on the inflammasome stimuli, cell type, and inhibition scheme utilized.

In our model, glycine and MM containing glycine dampened IL-1*β* and LDH release if nigericin was used as stimuli when interacting with the cells for an extended period before inflammasome activation, but not when inflammasome activation was promoted by *Δcap67* infection [69]. Another interesting feature presented in our inflammasome inhibition model was the accumulation of pro-IL-1*β* intracellularly in cells treated with CM35. It is well characterized that neither caspase-1 activation nor inhibition has any impact on pro-IL-1*β* intracellular levels in activated cells, indicating that IL-1*β* translation is stable regardless of whether it is subsequently cleaved [80]. One explanation for this stability of intracellular levels during caspase-1 inhibition is that pro-IL-1*β*, along with other inflammasome unrelated proteins, are present in the supernatant of necrotic cells undergoing NLRP3 activation [79]. On the other hand, changes in the intracellular cytokine reservoir can impact the secretion of its mature form [81]. In our work, an increase in pro-IL-1*β* intracellular levels occurred concomitantly with a decrease in IL-1*β* mature form release, which is not fully explained by the inhibition seen in caspase-1 activation. One aspect of our model is that pyroptosis is prevented in the presence of CM35, suggesting that intracellular proteins are retained during NLRP3 activation in this group. Furthermore, transcripts of *IL-1β* were highly expressed in the cells treated with CM35. Regarding inflammasome inhibition and pro-IL-1*β*, Folco et al. demonstrated that macrophages primed with LPS and treated with bafilomycin, a known inhibitor of the NLRP3-dependent receptor activation, exhibited increased intracellular levels of this cytokine [82].

The first studies depicting inflammasome activation by fungi demonstrated that morphogenesis is important to promote NLRP3 inflammasome activation; hence, some morphotypes of a pathogen can induce a more robust response than others [48, 83]. *C. neoformans* is also known to be a weak inflammasome activator. In this context, GXM has been considered the main factor for the yeast to evade inflammasome activation, promoting the evasion of phagocytosis and prevention of recognition by extracellular receptors [52–54].

The only well-described direct modulation of the inflammasome pathway to date with a focus on fungicidal activity has been with *C. albicans* internalized hyphae in which the secretion of a candidalysin leads to cell piercing, NLRP3 inflammasome activation, and host cell death via pyroptosis [84–87]. Despite the lack of further published evidence for fungal inhibition of inflammasome activation, many other intracellular pathogens, like bacteria and viruses, have well-described mechanisms for NLRP3 suppression. The human pathogenic bacteria of the genus *Yersinia* express a conserved type III secretion system (T3SS) as a virulence trait, which is associated with inflammasome activation. This secretion system is responsible for the release of effector proteins called *Yersinia* outer proteins (Yops), and two Yops can inhibit inflammasome activation: YopK is related to prevention of T3SS recognition by NLR receptors [88], and YopM is related to blockage of pyrin inflammasome activation [89]. Defects in both effector proteins lead to more robust inflammasome activation and bacterial clearance, highlighting the importance of the inhibition promoted by the pathogen. Poxviruses produce proteins homologous to mammalian proteins responsible for inflammasome inhibition, known as pyrin domain-only proteins (POP) and serpins. These viral proteins bind to ASC or caspase domains, preventing proper inflammasome assembly and activity from promoting the intracellular survival of the poxvirus [90]. Therefore, it is not surprising to demonstrate that *C. neoformans-*derived molecules can specifically inhibit inflammasome activity, and it is feasible to hypothesize that other fungal-driven mechanisms for inflammasome inhibition will be discovered soon.

While most studies have shown that IL-18 is important during cryptococcosis, IL-1*β* itself is sometimes depicted as unnecessary for host protection [55]. However, a recent study has shown that IL-1R signaling is essential for a Th1/Th17 polarization in a chronic infection model, consequentially facilitating fungal clearance by the host [56]. These data corroborate the importance of the NLRP3 components during cryptococcosis [52]. We demonstrate that macrophages treated with CM35 had a reduced capacity to control *C. neoformans*; the secretion of inflammasome-related cytokines is usually accompanied by cell death via pyroptosis. This cellular lysis releases a high content of proinflammatory IL-1 family cytokines, therefore activating neighboring cells thus promoting the maintenance of a proinflammatory environment [78]. Overall, inflammasome activation is related to a more effective response against *C. neoformans*, eliciting polarization towards Th1 and promoting antimicrobial macrophage activation; consequently, inhibition of this pathway reduces the responsiveness of the macrophages against the fungus.

The mechanisms and causes of vomocytosis are still poorly understood, but it is accepted that macrophages undergo vomocytosis because they cannot control their intracellular fungal burden [77]. Therefore, macrophages release the fungus to the extracellular environment instead of permitting themselves to serve as a protected niche for cryptococcal replication. Extracellular yeasts are less capable of evading the immune killing, and therefore, vomocytosis should lead to milder disease and is considered relatively protective [91]. Alanio and colleagues demonstrated that the outcome of human patients was highly correlated with the intracellular growth rate of virulent strains of *C. neoformans* [64], supporting this vomocytosis immune evasion hypothesis. Our results indicate that macrophages treated with CM35 have a higher rate of vomocytosis events, suggesting that the inhibition in the inflammatory response and pyroptosis promoted by the conditioned media might also enhance vomocytosis as an alternative mechanism to expel fungal burden to mitigate against host cell death.

ILA is an aromatic metabolite derived from the tryptophan pathway. It is produced by a wide variety of organisms and microorganisms, ranging from soil bacteria to humans [73, 74]. ILA production in fungi is mostly commonly seen in endophytic and phytopathogenic species and necessary for plant tissue colonization [92]. The tryptophan degradation pathway that leads to ILA production has an intermediate product denominated indolepyruvate, which is transformed in ILA as a result of a reduction-oxidation reaction by the NADPH-dependent enzyme indole-3-lactate dehydrogenase. Although this pathway and responsible enzymes have not yet been fully annotated in *C. neoformans* or *C. albicans*, the presence of ILA has been observed in conditioned media from both pathogenic yeasts [93]. Zelante et al. correlated the production of tryptophan catabolites by mouse gut microbiota, among then ILA, with mucosal protection from inflammation and resistance in a candidiasis infection model, which is mediated via IL-22 production [94].

In recent years, various enzymes and proteins related to biosynthesis metabolic pathways have been depicted as important for pathogenesis in cryptococcosis infection models, especially those associated with glucose metabolism. Defects in pyruvate and hexose kinases and in acetyl-CoA production impact virulence traits of the yeast, resulting in a reduction in host mortality [95, 96]. Nevertheless, our knowledge regarding aromatic metabolites derived from amino acid metabolism secreted by *C. neoformans* and their impact on the host during infection is severely limited. Notably, amino acid permeases are essential for the protection of the yeast when challenged by environmental or host-promoted stress conditions. *C. neoformans* mutants with a defect in these enzymes are less virulence, highlighting the importance of amino acid uptake during infection [97]. Yeast cells deficient in a small protein allegedly involved in the citric acid cycle had a higher expression of amino acids (i.e., tryptophan), and the mutant cells were more lethal in mice compared to wild-type yeast cells [98]. Another interesting aspect of this study was the demonstration that the intracellular fungal burden in macrophages infected with the mutant was only higher in the presence of exogenous NADPH, a cofactor that is essential to produce ILA from indol-3-pyruvate [86].

Although there is no report correlating aromatic metabolites and inflammasome inhibition, some small molecules with structures similar to aromatic metabolites are known to inhibit inflammasome. Glyburide is one of the most well-described inhibitors for NLRP3 activation, acting on ATP-sensitive potassium channels to block potassium efflux, which prevents inflammasome activation. Glyburide is a sulfonylurea drug with aromatic hydrocarbons in its structure [99]. Interestingly, not all sulfonylurea drugs are able to prevent IL-1*β* secretion via inflammasome inhibition, and there are reports of sulfonylurea drugs that prevent inflammasome activation by mechanisms other than potassium efflux blockage [100] which we hypothesize may be similar to the effects of ILA.

## 5. Conclusions

This work identifies new effects mediated by *C. neoformans* wild-type secreted molecules, especially in the ability to modify the activation of the intracellular inflammasome pathway. Treating macrophages with conditioned media reduced such critical functions such as phagocytosis and intracellular killing in *in vitro* infection models, suggesting a possible new mechanism for fungal persistence inside the host. We also found that the aromatic metabolite ILA mimics the inhibitory properties of CM35. Future studies regarding ILA and CM35 effects in fungal infection are essential to determine the detailed mechanism of action, to define where along the signaling pathway that the inflammasome is being affected and identifying other molecules that participate in inflammasome inhibition. An in-depth analysis of how these molecules impact cryptococcal infection would significantly enhance our understanding of cryptococcosis and perhaps lead to new strategies to prevent and treat *C. neoformans* disease.

## Data Availability

The data used to support the findings of this study are available from the corresponding author upon request.

## Abbreviations

ASC: Apoptosis-associated speck-like protein containing a CARD
BMDC: Bone marrow-derived dendritic cells with GM-CSF stimulus
BMDM: Bone marrow-derived macrophages with GM-CSF stimulus
BMM: Bone marrow-derived macrophages with M-CSF stimulus
CFU: Colony forming units
CM35: Conditioned media obtained from B3501 culture
CMCAP: Conditioned media obtained from *Δcap67* culture
ELISA: Enzyme-linked immunosorbent assay
GM-CSF: Granulocyte-macrophage colony-stimulating factor
GXM: Glucuronoxylomannan
HPLA: DL-p-Hydroxyphenyllactic acid
IL-18: Interleukin 18
IL-1*β*: Interleukin 1 beta
ILA: DL-Indole-3-lactic acid
kDa: Kilodalton
LDH: Lactate dehydrogenase
LPS: Lipopolysaccharide
mAb: Monoclonal antibody
M-CSF: Macrophage colony-stimulating factor
MM: Minimal media
NLRP3: NLR pyrin domain-containing 3
PBS: Phosphate-buffered saline
PLA: 3-Phenyllactic acid
PS: Polysaccharide
TNF-*α*: Tumor necrosis factor alpha.

## Supplementary Materials

Supplementary Materials S1 Fig: CM35, but not CMCAP or minimal media, is able to reduce IL-1*β* secretion. Secretion of IL-1*β* was measured from BMDMs stimulated with LPS (500 ng/ml) and nigericin (20 *μ*M), with or without possible inhibitor (10% *v*/*v*) overnight (18 h) (A). Secretion of TNF-*α* was measured from BMDMs (B), BMMs (C), and DCs (D) stimulated with LPS (500 ng/ml) and nigericin (20 *μ*M), with or without possible inhibitor (10% *v*/*v*) overnight (18 h). Alternatively, BMDMs were treated with possible inhibitors overnight (18 h) previously to stimulation with LPS (500 ng/ml) for 4 h (E). TNF-*α* release measured from supernatants of BMMs stimulated with nigericin (20 *μ*M), with or without CMCAP (F). Secretion of IL-1*β* (G) or TNF-*α* (H) was measured from BMMs stimulated with LPS (500 ng/ml) and nigericin (20 *μ*M), with or without possible inhibitor (10% *v*/*v*) overnight (18 h). Supernatants were collected after stimulus and cytokines measured by ELISA technique. CM35 = conditioned media from B3501; CMCAP = conditioned media from *Δcap67*; MM = minimal media; CM99 = conditioned media from H99. Statistical analysis was performed utilizing one-way ANOVA, where ns: not significant; ^∗^*P* ≤ 0.033; ^∗∗∗^*P* ≤ 0.001. S2 Fig: CMs induce IL-1*β* transcription in activated macrophages. Transcript levels of IL-1*β* (A) and Nf-*κ*B (B) from cDNA extracted from BMMs stimulated with LPS (500 ng/ml) and/or nigericin (20 *μ*M), with or without potential inhibitor (10% *v*/*v*) overnight (18 h). IL-1*β* or Nf-*κ*B to GAPDH relative expression was calculated using the 2^(-ct)^ method and normalized to the level of unstimulated BMMs. Statistical analysis was achieved by one-way ANOVA, where ns: not significant; ^∗^*P* ≤ 0.033; ^∗∗∗^*P* ≤ 0.001. Comparisons were made with the LPS+nigericin group. S3 Fig: GXM detection in CM samples. (A–C) BMMs interacting with inhibitors CM35 (A), CM35<1 kDa (B), and CMCAP (C). (D–F) BMMs interacting with CM35 inhibitor before GXM depletion treatment by capture ELISA (D), after treatment (E) and enriched with GXM eluted from the ELISA plate (F). Cells were stained for the nucleus (blue) and GXM (red). Images were taken in a confocal fluorescence microscope. S4 Fig: mass spectrometry comparative CM analysis. (A) Heat map based on extracted metabolite significant intensities from CM35 and CMCAP. (B–D) Comparative data from each aromatic metabolite detected in the analysis.
