# Supplementary material for: Cryptococcus neoformans Secretes Small Molecules That Inhibit IL-1β Inflammasome-Dependent Secretion
Source: Mediators Inflamm. 2020 Dec 3;2020:3412763. doi: 10.1155/2020/3412763 (PMC7748918; doi:10.1155/2020/3412763)
Supplement: Supplementary Materials — S1 Fig: CM35, but not CMCAP or minimal media, is able to reduce IL-1β secretion. Secretion of IL-1β was measured from BMDMs stimulated with LPS (500 ng/ml) and nigericin (20 μM), with or without possible inhibitor (10% v/v) overnight (18 h) (A). Secretion of TNF-α was measured from BMDMs (B), BMMs (C), and DCs (D) stimulated with LPS (500 ng/ml) and nigericin (20 μM), with or without possible inhibitor (10% v/v) overnight (18 h). Alternatively, BMDMs were treated with possible inhibitors overnight (18 h) previously to stimulation with LPS (500 ng/ml) for 4 h (E). TNF-α release measured from supernatants of BMMs stimulated with nigericin (20 μM), with or without CMCAP (F). Secretion of IL-1β (G) or TNF-α (H) was measured from BMMs stimulated with LPS (500 ng/ml) and nigericin (20 μM), with or without possible inhibitor (10% v/v) overnight (18 h). Supernatants were collected after stimulus and cytokines measured by ELISA technique. CM35 = conditioned media from B3501; CMCAP = conditioned media from Δcap67; MM = minimal media; CM99 = conditioned media from H99. Statistical analysis was performed utilizing one-way ANOVA, where ns: not significant; ∗P ≤ 0.033; ∗∗∗P ≤ 0.001. S2 Fig: CMs induce IL-1β transcription in activated macrophages. Transcript levels of IL-1β (A) and Nf-κB (B) from cDNA extracted from BMMs stimulated with LPS (500 ng/ml) and/or nigericin (20 μM), with or without potential inhibitor (10% v/v) overnight (18 h). IL-1β or Nf-κB to GAPDH relative expression was calculated using the 2(-ct) method and normalized to the level of unstimulated BMMs. Statistical analysis was achieved by one-way ANOVA, where ns: not significant; ∗P ≤ 0.033; ∗∗∗P ≤ 0.001. Comparisons were made with the LPS+nigericin group. S3 Fig: GXM detection in CM samples. (A–C) BMMs interacting with inhibitors CM35 (A), CM35<1 kDa (B), and CMCAP (C). (D–F) BMMs interacting with CM35 inhibitor before GXM depletion treatment by capture ELISA (D), after treatment (E) and enriched with GXM elute [file 3412763.f1.pdf]

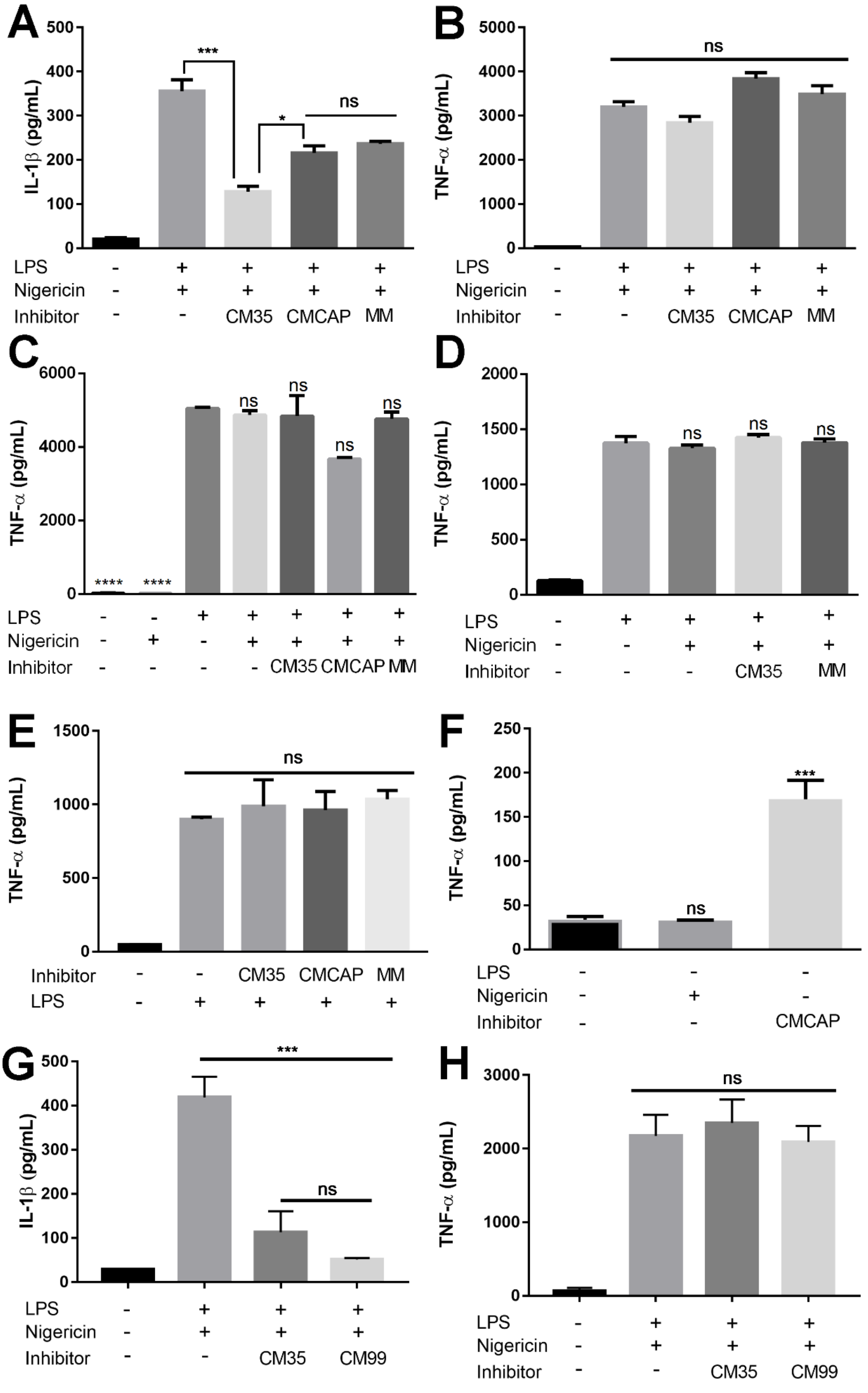

S1 Fig. CM35, but not CMCAP or Minimal Media is able to reduce IL-1β secretion.

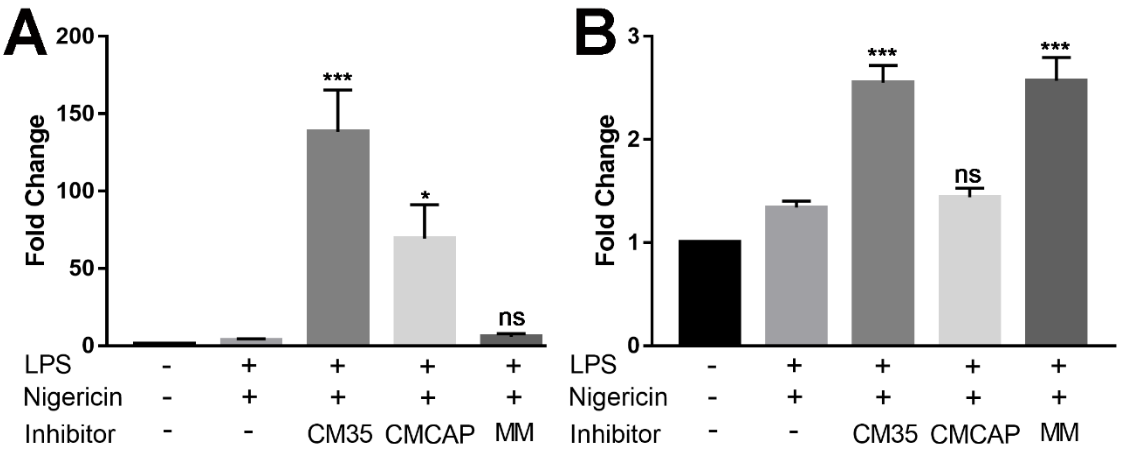

S2 Fig. CMs induce IL-1β transcription in activated macrophages.

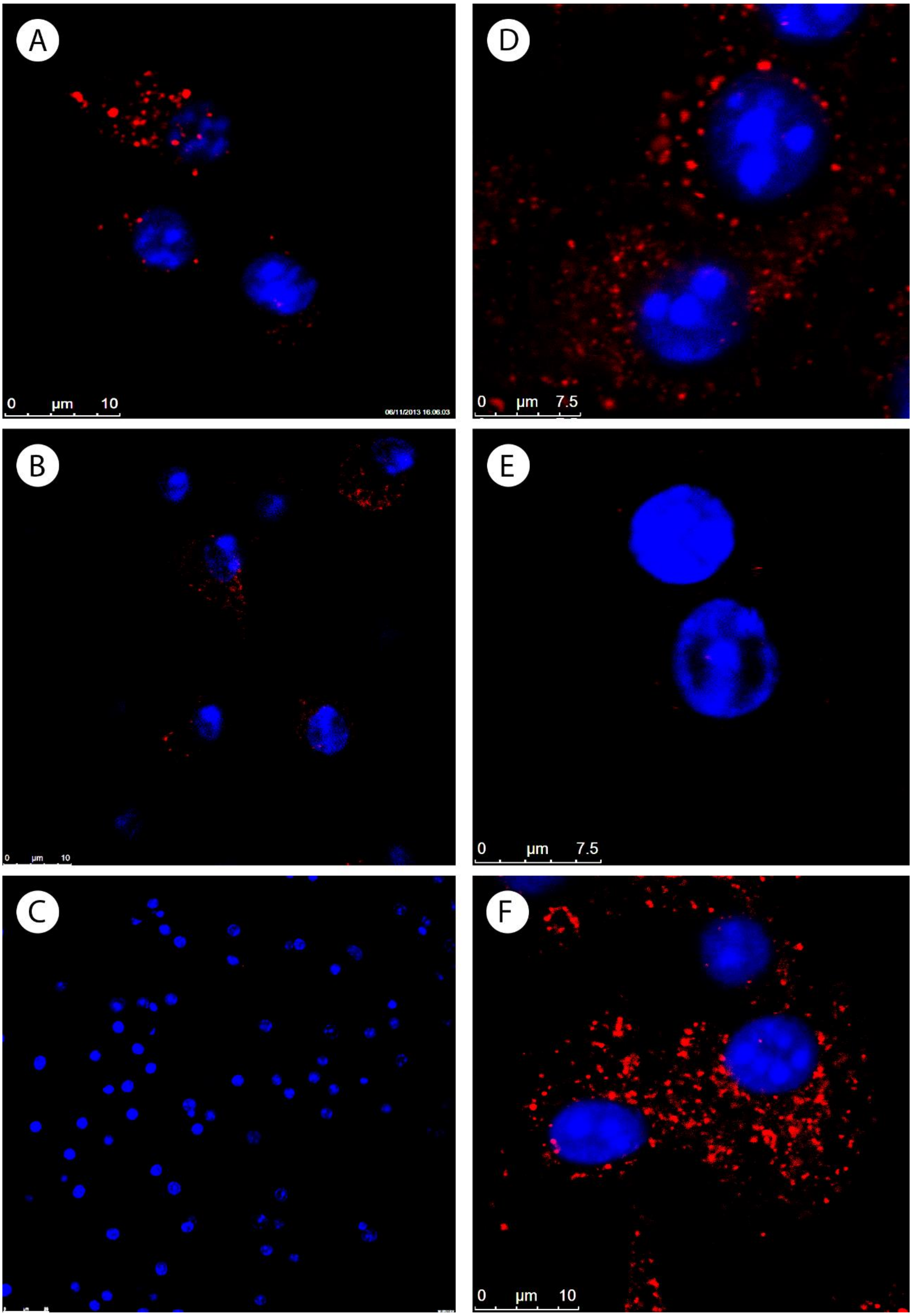

S3 Fig. GXM detection in CM samples.

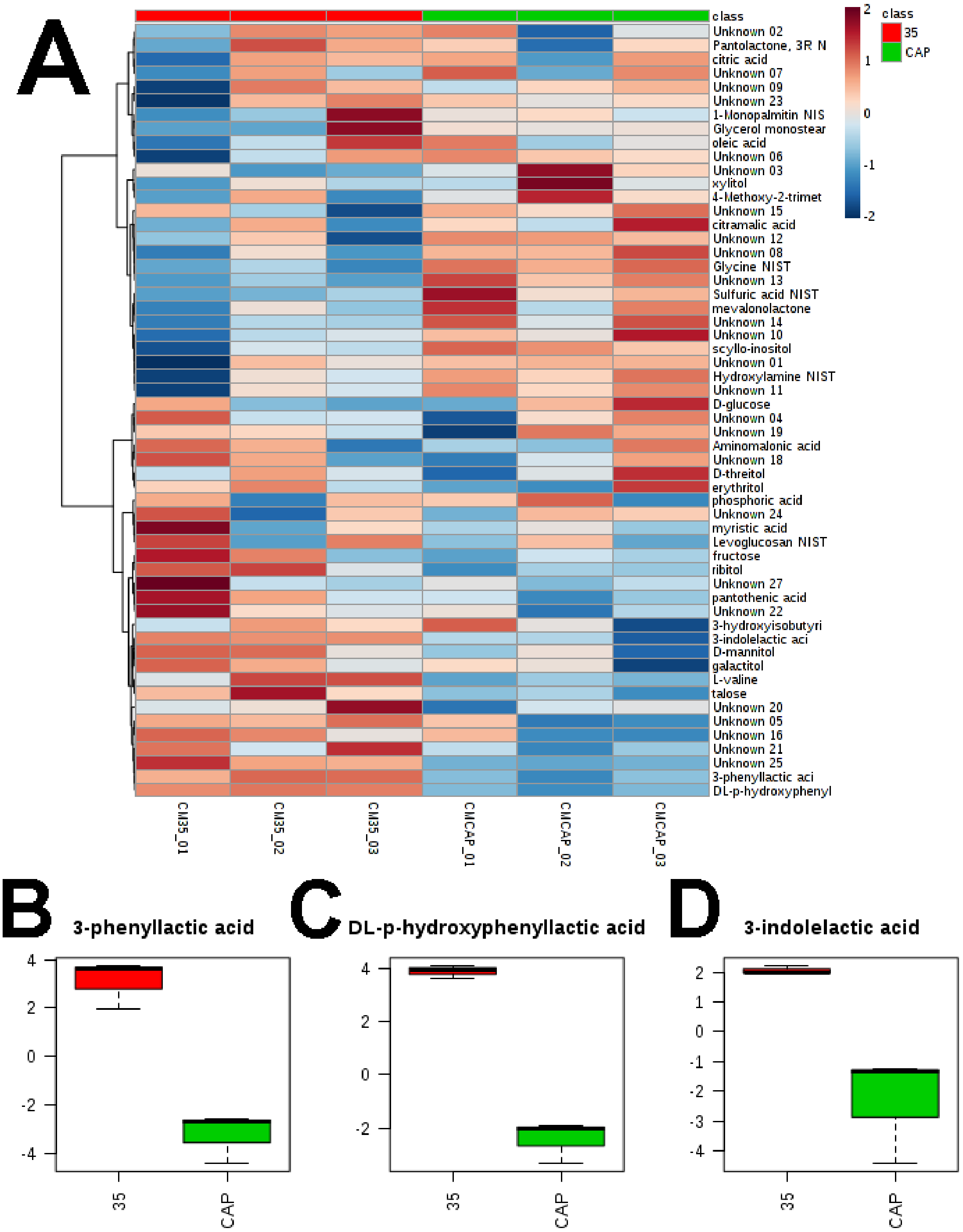

S4 Fig. Mass spectrometry comparative CM analysis.
